# Remarks on Proceedings of Southern Dental Association

**Published:** 1881-11

**Authors:** 


### EDITORIAL, ETC.

---

*Proceedings of the Southern Dental Association.*—The October number of the JOURNAL was filled completely with the Proceedings of the Southern Dental Association, as reported at its late meeting at Asheville, North Carolina, in July last. The writer undertook the task of editing these "Proceedings," and has done the best he could with the limited space at command. The printer was inexorable as to the number of pages, and no representation of the case could make him swell out the columns to sufficient length to take in all that was said. So the editor "boiled it all down," somewhat, though trying to omit nothing of importance. In any event, those who were present and who read the report will see that the writer "cut" *himself* more severely than any one else. But after all the matter ran over, and appears as a part of the November number. Again we beg our friends in the South to be liberal in their judgment, and deal lightly with the imperfections of the report. Some of the papers are very interesting, so we think, and will repay careful perusal. We may have more to say about this hereafter.

